# Behavioral and Emotional Difficulties and Personal Wellbeing of Adolescents in Residential Care

**DOI:** 10.3390/ijerph20010256

**Published:** 2022-12-24

**Authors:** Cristina Soriano-Díaz, Juan Manuel Moreno-Manso, María Elena García-Baamonde, Mónica Guerrero-Molina, Pilar Cantillo-Cordero

**Affiliations:** Department of Psychology, University of Extremadura, 06006 Badajoz, Spain

**Keywords:** mental health, emotional problems, behavioural problems, personal wellbeing, residential care, adolescents

## Abstract

This work studies the emotional and behavioural difficulties and the personal wellbeing of adolescents under protective measures. The sample is made up of 151 adolescents in residential care between 11 and 17 years of age. The instruments used were the Strengths and Difficulties Questionnaire (SDQ) and the Personal Wellbeing Index (PWI). The results indicate that a high percentage of adolescents have emotional and behavioural difficulties. We found a greater presence of behavioural rather than emotional problems in the adolescents. Similarly, we also found that females showed more emotional difficulties than the males. As for personal wellbeing, the results indicate that the adolescents are dissatisfied in several areas of their life, they feel insecure and have a pessimistic view of their future and of their achievements. Furthermore, those adolescents who were admitted to residential care due to something other than child abuse have a greater prosocial behaviour. It can be concluded that the more difficulties the adolescents experience (emotional, behavioural and with their peers), the lower the perception of their personal wellbeing will be. This study allows us to design interventions aimed at promoting psychological wellbeing among these adolescents.

## 1. Introduction

The family context, experiences and social interaction that occur in childhood are crucial in the emotional and social development of children and adolescents. When a child’s family context is adequate and the relationships are safe, it will develop a strong sense of belonging that will favour reaching maturity, emotional stability, and social development [1]. However, when the family circumstances and the relationships are conflictive and inappropriate and the experiences are adverse, the consequences will be reflected in the psychological wellbeing and mental health of the child [2,3].

Certain adverse life experiences in childhood, depending on the age at which they occur, their intensity, the significance they may have for the child and other circumstances that may surround the event, can have serious implications for the child’s psychological maturation. This is the situation of many children and adolescents who are in residential care due to their situation of vulnerability. To all this must be added the consequences of their separation of the family, the rupture of parental bonds when they enter residential childcare [4,5]; thus, the potential risk to these children and adolescents of suffering emotional and behavioural problems [6,7].

Residential care is a protection measure for those children and adolescents who, due to the severity of their situation, cannot remain in the family context and must be separated from the family. These minors may require therapeutic attention to face the possible emotional and behavioural repercussions derived from their situation of vulnerability, and thus they can achieve the psychological and emotional wellbeing they need.

Residential foster care in Spain is based on a specialized care model, as well as on the diversification of care resources according to the therapeutic needs of children and adolescents. In Spain the residential care service network is not similar in all regions. The current trend is to avoid residential care centers for all minors and to use specialized resources appropriate to the demands and needs of children and adolescents [8]. In Spain, the diversity of the profiles of the population cared for in the centers is increasing (minors who use violence against their parents, children and adolescents with mental health problems, unaccompanied foreign minors, minors with an advanced age that makes it difficult for them to be fostered with a family, minor offenders with protective measures), which requires specialized care [9].

Different studies have demonstrated that the presence of a psychopathology in children and adolescents in residential care is higher than that of the general population [10,11], showing different psychological disorders [12,13]. However, the residential care units that evaluate the mental health of minors are few and, if they do, only a small percentage of the children receive any therapeutic attention [14,15].

Cicchetti & Toth [16], Grasso et al. [17], and Moreno-Manso et al. [18] have provided evidence of the presence of emotional and behavioural problems in children and adolescents under protective measures, such as difficulties in facing social problems, impulsive, aggressive and defiant behaviour patterns, attention and information processing deficits, hyperactivity, anxiety symptoms, learning difficulties and problems with social skills necessary for evaluating the impact of their behaviour on others. 

Internalising and externalising behaviour are part of the behavioural and social adjustment of the children and adolescents, especially during adolescence. Nevertheless, this symptomatology in their extreme values can cause the presence of psychopathology [19], through which the adolescents will show such externalising behaviour patterns as disruptive conduct [20], aggressiveness [19,21], and rejection of rules and authority [19]. Similarly, there may also be internalising problems, such as problems with the regulation of emotions, depression and anxiety [5]. 

In this sense, there are various works of research that have pointed out the presence of both externalising and internalising behaviour patterns in children and adolescents in residential care [22,23]. Generally, it is the externalising problem that is at first most alarming and which is most easily visible. Valencia & Andrade [24] consider that this kind of conduct does not arise in an isolated manner, but that it is the result of the exteriorisation of a series of internal symptoms. 

Unfortunately, there are many children and adolescents with psychopathologies [25,26]. However, what is even more worrying is that many of them are not detected or do not receive therapeutic attention from the mental health services [11,14]. 

Likewise, various factors such as gender and time spent in residential care have been analyzed as variables that may intervene in the evolution of mental health problems presented by minors in residential care [27,28,29]. The behavioral and emotional problems of children and adolescents can be determined by the situation of vulnerability prior to residential care, or by long stay in residential care centers when it is not possible to provide an adequate therapeutic response to the needs of minors [30,31]. Differences in the mental health problems of children and adolescents according to gender have also been identified. Several studies have found more externalizing problems in males than in females [32,33]. For this reason, it is important that the research tries to identify the personal and contextual factors that may be involved in the mental health problems of minors [34].

Thus, research into the emotional and behavioural difficulties of children and adolescents under protective measures should be paramount [35]. Maher et al. [36] point to the need for residential care institutions to orient their decision-making towards the wellbeing of the children, taking on board the necessary evaluations at a cognitive, social, emotional and behavioural level, to be able to identify and provide adequate interventions to deal with the needs of these minors. 

Likewise, the evaluation of the personal wellbeing of adolescents under protective measures will allow us to know the subjective level of satisfaction and quality of life in relation to various specific areas, such as the family, health, life achievements, how safe a person feels, the groups to which they form a part, future security and relationships with other persons [37,38]. Individual differences in the perception of personal wellbeing of adolescents with adverse life experiences in childhood could determine the potential risk of suffering emotional and behavioural problems [39,40] and the detection of risk indicators could guide us regarding the support they need.

In Spain, several studies have been carried out that report on the subjective well-being of minors in residential care [41,42,43,44]. The studies showed lower subjective well-being in these adolescents. However, Casas & González-Carrasco [45] consider that it is necessary to take into account that adolescence can be a critical period for subjective well-being, given that, during this stage, satisfaction with life can decrease. Adolescence, together with experience if adverse experiences, family separation and entry into a residential care center, could intervene in the negative perception of well-being [46]. In addition to the above, the differences in subjective well-being according to gender is another aspect that has been dealt with in different studies with minors in residential care. Dinisman et al. [47], Llosada-Gistau et al. [43,44], González-García et al. [41] and Selwyn et al. [48] have found that there are significant differences according to gender, with subjective well-being being lower in females than in males. 

In this context, the objectives of this present study are: to analyse the emotional and behavioural problems and the personal wellbeing of adolescents under protective measures; to determine the presence of significant differences in emotional and behavioural difficulties and the personal wellbeing according to gender, protective measures and the time spent in residential care; to examine the relationship between emotional and behavioural problems and the perception of the personal wellbeing of adolescents; and to analyse the predictive value of personal wellbeing in the adolescents’ emotional and behavioural problems. Based on the theoretical review carried out, we expected that the adolescents would present emotional and behavioural difficulties and problems with their personal wellbeing (Hypothesis 1). Concerning gender, we also expected that the protective measures and the time spent in residential care would show significant differences in emotional and behavioural difficulties and in the perception of personal wellbeing (Hypothesis 2). In addition, we also considered that emotional and behavioural difficulties would be related to the adolescents’ perception of personal wellbeing (Hypothesis 3). Finally, we expected that the perception of personal wellbeing would act as a predictor of the adolescents’ emotional and behavioural difficulties (Hypothesis 4).

## 2. Method

### 2.1. Participants

The sample is composed of 151 adolescents in residential care in the Region of Extremadura (Spain). The participants were between 11 and 17 years of age (*M* = 14.36 years; *DT* = 1.853), 52.3% were female (*n* = 79) and 47.7% male (*n* = 71). As for time spent in residential care, the distribution is as follows: 66.2% of the adolescents (*n* = 100) had been in residential care for over 48 months and 33.8% (*n* = 51) less than 48 months (*M* = 46.23 months; *DT* = 34.35). The reason for the protective measure in 44.4% of the cases (*n* = 67) was abuse. As for the type of abuse, 16.5% had suffered physical neglect, followed by physical and emotional neglect (11.3%), physical abuse (9.3%), physical and emotional abuse (6%) and sexual abuse (1.3%). In the remaining 55.6% (*n* = 84), the protective measure was due to other reasons, such as the impossibility of the carers of fulfilling their parental obligations (32.4%), failure of parental control (19.2%) or the renunciation or abandonment of the parents (4%).

The participants were resident in various residential care centres for minors and tutored flats in the Region of Extremadura (Spain). Those cases under emergency measures or undergoing evaluation were not included in the research. Furthermore, the minimum period of residence in care was six months.

### 2.2. Instruments

The instruments used were the following:

1. Strengths and Difficulties Questionnaire, SDQ [49]. This is a screening instrument that allows the evaluation of emotional and behavioural difficulties, as well as of prosocial behaviour in childhood and adolescence, from a multi-informant perspective. The questionnaire has 25 items and is based on a dimensional model that evaluates five dimensions, each one valued through five items: Emotional Problems, Behavioural Problems, Hyperactivity, Problems with companions and Prosocial Conduct. The first four make up a total score for Difficulties. The scores of the five dimensions go from 0 to 10 and permit the classification of the absence or presence of Emotional Problems (*normal* = 0–5; *limit* = 6; *abnormal* > 6), Behavioural Problems (*normal* = 0–3; *limit* = 4; *abnormal* > 4), Hyperactivity (*normal* = 0–5; *limit* = 6; *abnormal* > 6), Problems with Companions (*normal* = 0–3; *limit* = 4–5; *abnormal* > 5), and Prosocial Conduct (*normal* = 6–10; *limit* = 5; *abnormal* = 0–4). Similarly, the total scale of Difficulties allows us to establish cut-off points (*normal* = 0–15; *limit* = 16-19; *abnormal =* 20–40). Internal consistency is α = 0.68 [49] and that found in our study is α = 0.77.
2. Personal Well-Being Index, PWI [50]. This is an instrument that evaluates the perception of personal wellbeing. The PWI is a scale that was designed as part of the Australian Unit Wellbeing Index and evaluated satisfaction in different areas of life, in a relatively generic and abstract way. In the study, we have used the adaptation of the PWI for adolescents by Casas et al. [38]. The test measures satisfaction in relation to 12 aspects of a person’s life: the family, health, standard of living, life achievements, how safe a person feels, the groups of which she/he forms a part, future security, relationships with other persons, leisure time, his/her own body, home life, and the degree of satisfaction with life, considered globally. The values range from “*completely dissatisfied*” to “*completely satisfied*” with scores from 0 to 10 and only the extremes are labelled corresponding to the highest scores at the highest levels of wellbeing. Internal consistency is α = 0.80 [50]. The internal consistency found in our study is α = 0.86.

### 2.3. Procedure

First, authorisation was requested from the institution (Region of Extremadura, Spain) responsible for the minors in loco parentis, as legal caregiver, to carry out the research. 

The instruments were applied in the residential care centres and tutored flats where the adolescents resided. As for the application of the instruments, the adolescents participated voluntarily in the research and agreement to complete the instruments was made individually. The instruments were applied at one-time point.

The research was authorised and approved by the University of Extremadura. All procedures performed were in accordance with the ethical standards of Extremadura University (Ref.: 181/2020) and with the 1964 Helsinki declaration and its later amendments or comparable ethical standards.

### 2.4. Data Analysis

We first carried out a descriptive analysis of the behavioural and emotional problems and of the perception of the personal wellbeing of the adolescents in residential care (Hypothesis 1). According to the central limit theorem, it was appropriate to use parametric tests in accordance with the nature of the variables and the sample size; we performed an inferential analysis to determine whether there were statistically significant differences in the behavioural and emotional problems and in the adolescents’ perception of their personal wellbeing with respect to gender, time spent in residential care and the reason for the protective measures (Hypothesis 2). To be more precise, we used the Welch t test to compare the means in the independent samples with unequal variances. 

Similarly, we also carried out a Pearson’s correlation analysis to analyse the relation between the emotional and behavioural problems and the perception of personal wellbeing (Hypothesis 3). We then performed a linear regression to determine the extent to which the perception of personal wellbeing can predict the adolescents’ emotional and behavioural difficulties (Hypothesis 4).

The package SPSS version 25 was used for the statistical treatment of the data.

## 3. Results

Table 1 shows the distribution of the participants in the responses to the Strengths and Difficulties Questionnaire (SDQ) and the Personal Wellbeing Index (PWI).

The results of the SDQ arrive at a normal score in the total difficulties scale, although the mean is close to the limit range (M = 15.97; SD = 6.27). As for the distribution of the sample, 39.1% of the adolescents in residential care have a normal score in the total difficulties scale, while 38.4% are in limit range and the remaining 22.5% are in the abnormal range. Compared to the normative data, we found that a high percentage of adolescents have behavioural problems (38.4%), hyperactivity (26.5%), emotional difficulties (23.8%) and, to a lesser extent, problems with their peers (17.9%). As for prosocial conduct, the results indicate that 13.9% of the adolescents are in the abnormal range (M = 7.09; SD = 2.25). 

The global personal wellbeing index of the PWI shows average scores among the adolescents (M = 61.17; SD = 13.18). Compared to the normative data [29], we find lower scores for personal wellbeing with respect to their lives (M = 5.30; SD = 1.79), security (M = 5.38; SD = 1.89), future (M = 5.41; SD = 1.99) and their achievements (M = 5.50; SD = 1.92). On the other hand, there is a greater perception of personal wellbeing with leisure time (M = 7.16; SD = 2.02), relationships with others (M = 7.14; SD = 2.01), the groups they are a part of (M = 6.99; SD = 2.01) and their health (M = 6.67; SD = 1.99).

To address the second hypothesis of the study, we carried out a comparative analysis of the means to find out whether there are differences, according to gender, the time spent in care and the reason for the protective measures, between the adolescents’ behavioural and emotional problems and their perception of personal wellbeing (Table 2 and Table 3).

The results indicate that there are significant differences depending on gender in emotional problems (*t* [149] = 4.07; *p* < 0.001), problems with companions (*t* [149] = 2.86; *p* = 0.005) and prosocial conduct (*t* [149] = 2.42; *p* = 0.017), as well as in the total difficulties scale (*t* [149] = 2.49; *p* = 0.014). The size (Cohen’s *d*) is 0.66, 0.46, 0.40 and 0.41, respectively, indicated a medium effect. The results demonstrate that the girls have more emotional problems, problems with companions and global difficulties than the boys, although we should take into consideration the fact that the girls also have higher scores in prosocial conduct.

As for the perception of personal wellbeing, it can be seen that there are significant differences in global personal wellbeing (*t* [149] = −3.31; *p* = 0.001; *d* = −6.69), family (*t* [149] = −2.11; *p* = 0.036; *d* = −0.83), standard of living (*t* [149] = −3.55; *p* = 0.001; *d* = −1.13), achievements (*t* [149] = −2.02; *p* = 0.045; *d* = −0.62), security (*t* [149] = −2.82; *p* = 0.005; *d* = −0.85), relationships (*t* [149] = −2.17; *p* = 0.032; *d* = −0.69), leisure (*t* [149] = −2.76; *p* = 0.007; *d* = −0.86), body (*t* [149] = −3.46; *p* = 0.001; *d* = −1.32) and life as a whole (*t* [149] = −2.55; *p* = 0.012; *d* = −0.72) with respect to the gender of the adolescents in residential care. In this sense, medium effect sizes (Cohen’s d) have been obtained in most of the spheres of personal wellbeing, except for the difference in the perception of personal wellbeing with respect to achievements, relationships with others and life considered globally, which all showed a small size effect. The data demonstrate that the boys have higher scores than the girls in their perception of personal wellbeing with respect to global personal wellbeing, the family, standard of living, life achievements, how safe they feel, their relations with others, leisure, their bodies, and life as a whole, considered globally.

As for the time spent in care, differences are only found in the perception of personal wellbeing with respect to how safe the adolescents feel (*t* [149] = −1.99; *p* = 0.048), which corresponds to a small effect size of Cohen (*d* = 0.36). Thus, we can see that those who have been in residential care for over two years feel safer.

Finally, there are significant differences in prosocial conduct with respect to the reason for being in care (*t* [149] = 2.11; *p* = 0.036). Those adolescents who were in care for other reasons than abuse had higher scores in prosocial conduct. In this sense, it should be noted that the effect size of the differences is small (*d* = 0.35).

As for the correlation analysis (Hypothesis 3), Table 4 shows that the total difficulties scale correlates negatively with global wellbeing (*p* < 0.001), the family (*p* = 0.017), health (*p* = 0.007), standard of living (*p* = 0.001), achievements (*p* = 0.002), security (*p* < 0.001), the group (*p* = 0.020), the future (*p* < 0.001), relationships (*p* = 0.017), leisure (*p* = 0.003), their body (*p* < 0.001) and life as a whole (*p* = 0.001).

As for emotional problems, they correlate negatively with global personal wellbeing (*p* < 0.001), standard of living (*p* = 0.008), achievements (*p* = 0.012), security (*p* = 0.002), the group (*p* = 0.030), the future (*p* = 0.037), relationships (*p* = 0.024), leisure (*p* = 0.001), their body (*p* = 0.001) and life as a whole (*p* = 0.001).

On the other hand, behavioural problems correlate negatively with global wellbeing (*p* = 0.001), health (*p* = 0.035), standard of living (*p* = 0.008), achievements (*p* = 0.025), security (*p* = 0.018), the future (*p* = 0.004), their body (*p* = 0.027) and life as a whole (*p* = 0.005).

Hyperactivity shows negative correlations with global wellbeing (*p* = 0.031), security (*p* = 0.007), the future (*p* = 0.002) and their body (*p* = 0.014)0. The results demonstrate that the greater the hyperactivity, the lower the perception of personal wellbeing in the adolescents at a global level, as well as in security, the future, and their body.

Finally, it can be stated that problems with companions correlate negatively with global wellbeing (*p* < 0.001), the family (*p* = 0.037), health (*p* = 0.011), standard of living (*p* = 0.033), achievements (*p* = 0.005), the group (*p* < 0.001), the future (*p* = 0.009), relationships (*p* = 0.049), leisure (*p* = 0.003), their body (*p* = 0.006) and life as a whole (*p* = 0.036).

Finally, for our fourth hypothesis, we carried out a regression analysis to determine the extent to which the perception of personal wellbeing can significantly predict the emotional and behavioural problems of adolescents in residential care (Table 5 and Table 6).

Personal wellbeing scales make it possible to negatively predict difficulties. Therefore, global personal wellbeing explains 13.2% of the variance in the responses concerning difficulties, 3.8% concerning the family, 4.7% for health, 6.6% for standard of living, 6% for achievements, 8.8% for security, 3.6% for the group, 9.6% for the future, 3.7% for relationships, 6% for leisure, 9.6% for the body and 7.4% for life as a whole.

In the same way, the results indicate that personal wellbeing negatively predicts emotional problems. Global wellbeing explains 8.7% of the variance in the adolescents’ emotional problems, 4.6% for standard of living, 4.1% for achievements, 6.5% for security, 3.1% for the group, 2.9% for the future, 3.4% for relationships, 7% for leisure, 6.8% for the body and 5.5% for life as a whole.

As for negatively predicting the adolescents’ behavioural problems, the results allow us to state that global wellbeing explains 7% of the variance in behavioural problems, 3% for health, 4.6% for standard of living, 3.3% for achievements, 3.7% for security, 5.4% for the future, 3.2% for the body and 5.1% for life as a whole.

Similarly, global wellbeing, security, the future, and the body can negatively predict hyperactivity. In this sense, it is important to note that global personal wellbeing explains 3.1% of the variance in hyperactivity, 4.8% for security, 6.1% for the future and 4% for the body.

Finally, personal wellbeing scales negatively predict problems with companions. The results show that global personal wellbeing explains 8.5% of the variance in the responses concerning problems with companions, 2.9% for the family, 4.2% for health, 3% for standard of living, 5.1% for achievements, 11.4% for the group, 4.5% for the future, 2.6% for relationships, 5.6% for leisure, 4.9% for the body and 2.9% for life as a whole.

We can confirm that the adolescents’ personal wellbeing tends to significantly predict the existence of less behavioural and emotional problems, although it does not contribute to predicting the prosocial behaviour of adolescents in residential care.

## 4. Discussion

Based on the results of the study, we can conclude that there are adolescents under protective measures who have emotional and behavioural difficulties or are on the limit, according to the normative data. The prevalence of adolescents within the limit range should give us warning of the risk of suffering externalising and internalising problems in the future. Similarly, there are studies which have demonstrated that adolescents in residential care have a higher risk of suffering emotional and/or behavioural problems than the general population [6,51].

Our research has also found a greater presence of behavioural problems in these adolescents than emotional difficulties. These results agree with the conclusions of studies carried out by González-García et al. [22], Martín et al. [52]. and Moreno-Manso et al. [18]. Nevertheless, Delgado [53] and Fonseca-Pedrero et al. [54] found a higher prevalence of emotional problems in adolescents.

As for prosocial conduct, we can conclude that only a small percentage of adolescents are to be found within the abnormal range in this sub-scale. In general, the adolescents perceive themselves as having adequate competences to handle interactions with others, these being fundamental for personal wellbeing. Prosocial behaviour could act as a protective factor for future emotional and behavioural problems. Adolescents with prosocial conduct have a lower probability of experiencing behavioural [55] or emotional [56] difficulties. In our study, the adolescents expressed dissatisfaction with various areas of their lives, feeling insecure and with a pessimistic view of their future and their achievements in life; however, they had a positive view of their relationships with other persons and the groups to which they belonged. The sphere of relationships has been preserved, so the personal wellbeing index is situated around the average. The perception of prosocial conduct is related to personal wellbeing [57]. Relationships and the sense of belonging to a group are beneficial for psychological wellbeing and can prevent emotional problems [58].

As for the presence of significant differences according to gender, we found that the females had more emotional difficulties, problems with companions and difficulties on a global level than the males. Rodrigues et al. [59] and Schmid et al. [60] also found a greater presence of emotional problems in females, yet they also concluded that there was a greater presence of behavioural problems in males. In our study, although the presence of behavioural problems is greater in the males than in the females, no significant differences were observed. With respect to personal wellbeing on a global level, we found that the males had a better perception than the females of their family, relationships, ways of amusing themselves, their lives in general, their achievements, how secure they felt, and their bodies.

As for the time spent in residential care centres, no differences were found among the adolescents, with the exception that the adolescents who had been in care for over two years, as part of their perception of personal wellbeing, felt more secure than those who had spent less time in care. As Schütz et al. [61] pointed out, these results could be explained by the instability that many of these adolescents suffer, with continuous admissions and changes of residence.

No significant differences were found in emotional and behavioural problems either, or in the adolescents’ perception of their personal wellbeing according to protective measures. Nevertheless, the adolescents who were victims of child abuse showed a lower level of prosocial conduct. Unlike in our study, other studies did show evidence of a higher prevalence of emotional and behavioural problems in minors in care who were also victims of child abuse [59,62].

The research has also demonstrated the relation between emotional and behavioural problems and the adolescents’ perception of their personal wellbeing. The greater the difficulties they have (emotional, behavioural, hyperactivity and with companions), the lower their perception of personal wellbeing in general, as well as with respect to the family, health, standard of living, achievements in life, how secure they feel, the group they belong to, their future, their body, their relationships with others, their leisure time and life taken as a whole, globally. As pointed out by various studies, adverse experiences influence the perception of their personal wellbeing [63].

As for the limitations of this study, we should point out that the research is of a transversal character and that it is therefore situated within a particular temporal point of the adolescents’ lives. A longitudinal study could provide even better evidence of emotional and behavioural difficulties and problems in the adolescents’ perception of their personal wellbeing, from the moment they are admitted to a residential care centre. Similarly, given that the institutionalisation of these adolescents was motivated by different circumstances, it would be useful to analyse in greater detail the adverse conditions that led to their admittance to care. This is a limitation, since there are adolescents in residential care who, in addition to being in a situation of vulnerability (abuse, resignation, abandonment, impossibility of parental care), have problems of coexistence and family conflict (absence of parental control). Therefore, it is possible that some adolescents presented emotional and behavioural problems as a result of their situation of vulnerability, and in other adolescents their psychological symptomatology may have contributed to the reason that they required residential care. A comprehensive evaluation of these adolescents’ previous adverse childhood experiences would have strengthened the study. However, given that their admittance to a care centre did not include a psychological evaluation, it has not been possible to determine whether the symptomatology they present is prior to their admittance to the institution or a consequence of it. Finally, future research should consider adding covariates, such as demographic characteristics, to control regression models.

## 5. Conclusions

The research concludes that the adolescents’ perception of personal wellbeing tends to significantly predict the existence of behavioural and emotional problems. Similarly, Huebner et al. [64] concluded that wellbeing and satisfaction with life could prevent the appearance of future psychopathological problems in children and adolescents. The influence of psychological wellbeing on one’s general health seems to be clear, especially as a protective factor; yet, in addition, we know that it encourages a positive emotional control, an adequate cognitive functioning and good social development [65]. In a similar way, it also tends to influence behavioural and emotional problems, helping to prevent the possible risks of such problems appearing [66].

As a contribution to the study, we must point out that the results inform if the need for skilled supports to address the principal difficulties observed. We consider that support should focus on improving cognitive, emotional, and behavioural resources in the search for alternative solutions to resolve the day-to-day problems and to set up measures aimed at promoting adolescents’ wellbeing. To do so, it is fundamental to train them in real situations and natural contexts in which the adolescents are normally involved. We consider it relevant that adolescents learn competent social and emotional behaviors and develop personal resources to face daily life and the transition to adult life, from a perspective that facilitates wellbeing. Encouragement should be given towards a commitment to clearly established rules and routines that provide a sense of security and predictability in the environment (consistency and flexibility in the environment), so important to provide personal wellbeing. Training should be given in reflection and analysis skills that help them exert control over behavior and emotions and make decisions. Support should be given in understanding and managing emotions and the feelings that cause them, which can improve the perception of personal wellbeing and improve self-esteem. It will be necessary to carry out important educational work regarding the affective bonds of adolescents and their personal relationships.

Emotional and behavioural difficulties in care have important consequences for mental health, but also at an adaptive level. Many of these adolescents have difficulties facing adverse circumstances, are not very decisive, manage their emotions badly and lack an understanding of others’ emotions, which in turn influences the perception of their personal wellbeing. The most important factor in personal wellbeing is the quality of support and care, even more so than the care environment itself [67,68]. We trust that this research may serve to encourage future research into this subject in greater depth.

## Figures and Tables

**Table 1:** Descriptive statistics of the emotional and behavioural difficulties and of the adolescents’ personal wellbeing.

Variables	Normal Range		Limit Range		Abnormal Range		M	*SD*
	*n*	%	*n*	%	*n*	%		
Total Difficulties Scale	59	39.1	58	38.4	34	22.5	15.97	6.27
Emotional Problems	81	53.6	34	22.5	36	23.8	4.45	2.37
Behavioural Problems	62	41.1	31	20.5	58	38.4	3.56	2.22
Hyperactivity	73	48.3	38	25.2	40	26.5	4.90	2.50
Problems with Companions	84	55.6	40	26.5	27	17.9	3.07	1.91
Prosocial Conduct	107	70.9	23	15.2	21	13.9	7.09	2.25
Global Personal Wellbeing	---						61.17	13.18
Family	---						5.77	2.47
Health	---						6.67	1.99
Standard of living	---						6.02	2.07
Achievements	---						5.50	1.92
Security	---						5.38	1.89
Group	---						6.99	2.01
Future	---						5.41	1.99
Relationships	---						7.14	2.01
Leisure	---						7.16	2.02
Body	---						6.49	2.47
Centre	---						5.58	2.38
Whole life	---						5.30	1.79

**Table 2:** Comparison of the means of the difficulties and personal wellbeing according to gender and the time spent in care.

	Female		Male			Less than 2 Years		More than 2 Years		
	*M*	*SD*	*M*	*SD*	*t*	*M*	*SD*	*M*	*SD*	*t*
Difficulties	17.18	5.73	14.65	6.60	2.49 *	16.37	6.23	15.77	6.31	0.56
Emotional Problems	5.16	2.16	3.67	2.34	4.07 ***	4.59	2.25	4.38	2.43	0.52
Behavioural Problems	3.51	2.03	3.61	2.42	−2.86	3.75	2.39	3.46	2.13	0.72
Hyperactivity	5.03	2.33	4.76	2.69	0.526	4.84	2.31	4.93	2.61	−0.21
Problems with Companions	3.48	1.89	2.61	1.85	2.86 **	3.20	1.94	3.00	1.91	0.59
Prosocial Conduct	7.51	2.04	6.63	2.39	2.42 *	7.00	2.31	7.13	2.23	−0.33
Global Wellbeing	57.98	16.09	64.67	7.66	−3.31 **	60.26	12.31	61.64	13.63	−0.63
Family	5.38	2.85	6.21	1.91	−2.11 *	5.75	2.39	5.79	2.53	−0.11
Health	6.47	2.28	6.89	1.61	−1.32	6.53	1.81	6.74	2.08	−0.64
Standard of Living	5.48	2.41	6.61	1.41	−3.55 **	6.22	1.70	5.92	2.34	0.90
Achievements	5.20	2.19	5.82	1.53	−2.02 *	5.65	1.83	5.42	1.98	0.70
Security	4.97	2.04	5.82	1.62	−2.82 **	4.96	1.78	5.59	1.93	−1.99 *
Group	6.78	2.36	7.21	1.52	−1.32	6.84	2.02	7.06	2.01	−0.62
Future	5.38	2.17	5.44	1.79	−0.21	5.51	1.79	5.36	2.09	0.46
Relationships	6.81	2.30	7.50	1.56	−2.17 *	7.00	1.66	7.21	2.17	−0.66
Leisure	6.75	2.48	7.61	1.20	−2.76 **	7.02	1.89	7.23	2.08	−0.62
Body	5.86	2.82	7.18	1.79	−3.46 **	6.35	2.39	6.56	2.51	−0.49
Centre	5.53	2.46	5.64	2.29	−0.28	5.25	2.64	5.75	2.23	−1.15
Whole Life	4.96	2.08	5.68	1.33	−2.55 *	5.24	1.97	5.34	1.71	−0.32

Note: * *p* < 0.05; ** *p* < 0.01; *** *p* < 0.001.

**Table 3:** Comparison of the means of the difficulties and personal wellbeing according to the reason for the protective measures.

	Other Reasons		*Abuse*		
	*M*	*SD*	*M*	*SD*	*t*
Difficulties	15.99	5.86	15.96	6.79	0.31
Emotional Problems	4.51	2.37	4.37	2.37	0.36
Behavioural Problems	3.44	2.14	3.70	2.33	−0.71
Hyperactivity	4.98	2.46	4.81	2.57	0.41
Problems with Companions	3.06	1.85	3.07	2.01	−0.05
Prosocial Conduct	7.43	2.22	6.66	2.24	2.11 *
Global Wellbeing	61.30	13.75	61.01	12.53	0.14
Family	5.92	2.38	5.60	2.59	0.78
Health	6.82	1.97	6.48	2.02	1.05
Standard of Living	5.96	2.15	6.09	1.97	−0.37
Achievements	5.42	1.98	5.60	1.86	−0.57
Security	5.49	1.89	5.24	1.90	0.80
Group	7.12	1.77	6.82	2.28	0.88
Future	5.55	1.94	5.24	2.05	0.94
Relationships	7.14	2.11	7.13	1.88	0.03
Leisure	7.14	2.17	7.18	1.82	−0.11
Body	6.46	2.60	6.52	2.30	−0.14
Centre	5.43	2.52	5.78	2.18	−0.91
Whole Life	5.12	1.98	5.54	1.50	−1.47

Note: * *p* < 0.05.

**Table 4:** Correlations between the adolescents’ emotional and behavioural difficulties and their personal wellbeing.

	Difficulties	Emotional Problems	Behavioural Problems	Hyperactivity	Problems with Companions	Prosocial Conduct
Global Wellbeing	−0.364 ***	−0.295 ***	−0.264 **	−0.176 *	−0.292 ***	0.075
Family	−0.194 *	−0.128	−0.129	−0.121	−0.170 *	0.015
Health	−0.217 **	−0.129	−0.172 *	−0.111	−0.206 *	0.122
Standard of Living	−0.257 **	−0.216 **	−0.214 **	−0.118	−0.174 *	0.151
Achievements	−0.246 **	−0.203 *	−0.182 *	−0.089	−0.226 **	0.005
Security	−0.296 ***	−0.255 **	−0.193 *	−0.220 **	−0.145	0.044
Group	−0.190 *	−0.177 *	−0.087	0.028	−0.338 ***	0.036
Future	−0.310 ***	−0.170 *	−0.233 **	−0.247 **	−0.212 **	0.023
Relations	−0.194 *	−0.183 *	−0.157	−0.050	−0.160 *	0.148
Leisure	−0.244 **	−0.265 **	−0.154	−0.043	−0.238 **	−0.013
Body	−0.310 ***	−0.261 **	−0.180 *	−0.200 *	−0.221 **	−0.104
Centre	−0.061	−0.043	−0.104	−0.033	0.016	0.130
Life as a whole	−0.272 **	−0.234 **	−0.225 **	−0.128	−0.171 *	0.028

Note: * *p* < 0.05; ** *p* < 0.01; *** *p* < 0.001.

**Table 5:** Linear regression analysis between the adolescents’ emotional and behavioural difficulties and their personal wellbeing (I).

	Difficulties				Emotional Problems				Behavioural Problems			
	*R*²	β	IC 95%		*R*²	β	IC 95%		*R*²	β	IC 95%	
Global Wellbeing	0.132	−0.364 ***	−0.245	−0.101	0.087	−0.295 ***	−0.081	−0.025	0.070	−0.264 **	−0.071	−0.018
Family	0.038	−0.194 *	−0.895	−0.090	0.016	−0.128	−0.276	0.031	0.017	−0.129	−0.260	0.029
Health	0.047	−0.217 **	−1.180	−0.185	0.017	−0.129	−0.344	0.037	0.030	−0.172 *	−0.369	−0.014
Standard of living	0.066	−0.257 **	−1.253	−0.305	0.046	−0.216 **	−0.427	−0.066	0.046	−0.214 **	−0.399	−0.060
Achievements	0.060	−0.246 **	−1.312	−0.290	0.041	−0.203 *	−0.445	−0.055	0.033	−0.182 *	−0.394	−0.026
Security	0.088	−0.296 ***	−1.491	−0.468	0.065	−0.255 **	−0.513	−0.123	0.037	−0.193 *	−0.412	−0.040
Group	0.036	−0.190 *	−1.090	−0.096	0.031	−0.177 *	−0.397	−0.021	0.008	−0.087	−0.274	0.083
Future	0.096	−0.310 ***	−1.461	−0.491	0.029	−0.170 *	−0.391	−0.012	0.054	−0.233 **	−0.435	−0.084
Relations	0.037	−0.194 *	−1.101	−0.109	0.034	−0.183 *	−0.404	−0.028	0.025	−0.157	−0.350	0.004
Leisure	0.060	−0.244 **	−1.247	−0.271	0.070	−0.265 **	−0.494	−0.128	0.024	−0.154	−0.345	0.007
Body	0.096	−0.310 ***	−1.178	−0.396	0.068	−0.261 **	−0.400	−0.100	0.032	−0.180 *	−0.305	−0.019
Centre	0.004	−0.061	−0.588	0.265	0.002	−0.043	−0.204	0.118	0.011	−0.104	−0.247	0.054
Life as a whole	0.074	−0.272 **	−1.495	−0.405	0.055	−0.234 **	−0.516	−0.101	0.051	−0.225 **	−0.475	−0.084

Note: * *p* < 0.05; ** *p* < 0.01; *** *p* < 0.001.

**Table 6:** Linear regression analysis between the adolescents’ emotional and behavioural difficulties and their personal wellbeing (II).

	Hyperactivity				Problems with Companions				Prosocial Conduct			
	*R*²	β	IC 95%		*R*²	β	IC 95%		*R*²	β	IC 95%	
Global Wellbeing	0.031	−0.176 *	−0.064	−0.003	0.085	−0.292 ***	−0.065	−0.020	0.006	0.075	−0.015	0.040
Family	0.015	−0.121	−0.285	0.040	0.029	−0.170 *	−0.255	−0.008	0.000	0.015	−0.133	0.162
Health	0.012	−0.111	−0.341	0.063	0.042	−0.206 *	−0.350	−0.045	0.015	0.122	−0.044	0.320
Standard of living	0.014	−0.118	−0.337	0.052	0.030	−0.174 *	−0.308	−0.013	0.023	0.151	−0.010	0.339
Achievements	0.008	−0.089	−0.326	0.093	0.051	−0.226 **	−0.382	−0.068	0.000	0.005	−0.183	0.196
Security	0.048	−0.220 **	−0.498	−0.081	0.021	−0.145	−0.308	0.016	0.002	0.044	−0.140	0.244
Group	0.001	0.028	−0.167	0.236	0.114	−0.338 ***	−0.468	−0.177	0.001	0.036	−0.142	0.222
Future	0.061	−0.247 **	−0.508	−0.114	0.045	−0.212 **	−0.356	−0.052	0.001	0.023	−0.157	0.210
Relations	0.003	−0.050	−0.264	0.139	0.026	−0.160 *	−0.305	−0.001	0.022	0.148	−0.014	0.346
Leisure	0.002	−0.043	−0.254	0.147	0.056	−0.238 **	−0.375	−0.076	0.000	−0.013	−0.196	0.166
Body	0.040	−0.200 *	−0.364	−0.042	0.049	−0.221 **	−0.294	−0.049	0.011	−0.104	−0.242	0.052
Centre	0.001	−0.033	−0.205	0.136	0.000	0.016	−0.117	0.144	0.017	0.130	−0.029	0.276
Life as a whole	0.016	−0.128	−0.403	0.045	0.029	−0.171 *	−0.353	−0.012	0.001	0.028	−0.168	0.239

Note: * *p* < 0.05; ** *p* < 0.01; *** *p* < 0.001.
